# The plant-specific histone residue Phe41 is important for genome-wide H3.1 distribution

**DOI:** 10.1038/s41467-018-02976-9

**Published:** 2018-02-12

**Authors:** Li Lu, Xiangsong Chen, Shuiming Qian, Xuehua Zhong

**Affiliations:** 1 Laboratory of Genetics, University of Wisconsin-Madison, Madison, WI 53706 USA; 2 Wisconsin Institute for Discovery, University of Wisconsin-Madison, Madison, WI 53706 USA

## Abstract

The dynamic incorporation of histone variants influences chromatin structure and many biological processes. In *Arabidopsis*, the canonical variant H3.1 differs from H3.3 in four residues, one of which (H3.1Phe41) is unique and conserved in plants. However, its evolutionary significance remains unclear. Here, we show that Phe41 first appeared in H3.1 in ferns and became stable during land plant evolution. Unlike H3.1, which is specifically enriched in silent regions, H3.1F41Y variants gain ectopic accumulation at actively transcribed regions. Reciprocal tail and core domain swap experiments between H3.1 and H3.3 show that the H3.1 core, while necessary, is insufficient to restrict H3.1 to silent regions. We conclude that the vascular-plant-specific Phe41 is critical for H3.1 genomic distribution and may act collaboratively with the H3.1 core to regulate deposition patterns. This study reveals that Phe41 may have evolved to provide additional regulation of histone deposition in plants.

## Abstract (web summary)

The canonical histone variant H3.1 of vascular plants contains a conserved Phe residue at position 41 that is unique to the plant kingdom. Here, Lu et al. provide evidence that H3.1Phe41 acts collaboratively with the H3.1 core domain to restrict H3.1 deposition to silent regions of the genome.

## Introduction

The nucleosome is a fundamental unit of chromatin that packages genomic DNA in eukaryotes. Each nucleosome consists of 146–147 bp of DNA tightly wrapped by a core of eight histone proteins comprised of two H2A-H2B dimers and a H3-H4 tetramer^1,2^. All core histones, with the exception of H4, exist as protein variants that differ in a few amino acids^3,4^. Histone H3 can be classified into several subtypes. Besides the centromere-specific variant CenH3^5^ and testis-specific variant H3t^6^, H3 variants can be further divided into two groups based on their expression and incorporation dynamics^7,8^. H3.1 is the canonical variant that is predominately expressed in the S phase and incorporates into chromatin in a DNA replication-dependent manner, whereas the H3.3 variant is expressed and deposited via a DNA replication-independent pathway throughout the cell cycle^8,9^. The dynamic incorporation of histone variants influences nucleosome properties and plays an important role in DNA replication, transcription, recombination, and repair^10–12^. The spatial and temporal depositions of histone variants are strictly regulated by histone chaperones^8,10,13^. In addition, differences in the post-translational modifications (PTMs) between H3.1 and H3.3 provide another mechanism for their distinct chromatin functionality^7,9,14,15^.

Genome-wide profiling of *Drosophila* and mammalian H3 variants showed that H3.3 is mostly enriched in actively transcribed regions including the gene body of active genes, the promoters of both active and inactive genes, and some regulatory elements^16–18^. H3.3 is also found at certain heterochromatic loci in centromeres and telomeres in mouse embryonic stem cells^19,20^. Unlike animal H3.3, plant H3.3 has not been detected in the centromeric regions. *Arabidopsis* H3.3 is enriched at euchromatic regions, mostly in the gene body and promoter regions^21–23^. H3.3 abundance is correlated with gene expression levels^21,23^ and the presence of RNA polymerase II^22^. Additionally, *Arabidopsis* H3.3 is enriched at telomeres^24^. In contrast, H3.1 enrichment was found mostly at repetitive pericentromeric heterochromatin and is negatively correlated with gene expression levels^21,23^. Consistently, both genomic and mass spectrometry studies have revealed that H3.1-enriched regions are marked with silencing-related DNA methylation and histone modification marks (e.g., H3K9me2 and H3K27me1)^21,23,25,26^.

Despite the distinct deposition pattern and function, H3.3 and H3.1 differ in a surprisingly small number of amino acids. In *Drosophila*, four residues distinguish H3.3, featuring S31-A87-I89-G90, from H3.1, containing A31-S87-V89-M90^27^. Three of these residues are found within the core histone folding domain. Mutational analysis revealed that a single amino acid substitution at any of these three positions in H3.1 toward H3.3 allows replication-independent deposition in *Drosophila* cells, suggesting that the histone core region is central for its deposition^27^. Interestingly, while the N-terminal tail appears to be important for H3.1 deposition, H3.3 chromatin incorporation does not require its N-terminal tail^27^. Unlike animals, plant H3.3 and H3.1 typically differ in residues 31, 41, 87, and 90^28,29^. Similarly, fluorescence microscopy images showed that residues H87 and L90 in the core domain of *Arabidopsis* H3.3 are critical for its deposition into ribosomal DNA loci^28^. Besides the core regions, plant H3.3 differs from H3.1 with additional residues at positions 31 and 41, which lie in the H3 N-terminal tail. Recently, alanine 31 (Ala31) of H3.1 has been reported to mediate selective histone 3 lysine 27 mono-methylation at H3.1^15^. While the difference at position 31 is conserved between plants and animals, the position 41 difference is vascular-plant-specific (Phe41 in H3.1 versus Tyr41 in H3.3). Phylogenetic analysis further showed that Phe41 is a unique H3.1 feature that is conserved in vascular plants^30,31^, suggesting that H3.1 may have evolved independently in animals and plants. Despite its conservation, the evolutionary significance and function of vascular-plant-specific H3.1Phe41 has yet to be investigated.

Here, we utilized a combination of molecular, cellular, and genomic approaches to investigate the evolution and function of Phe41 in plants. We noted that Phe41 first appeared in H3.1 in ferns and became stable during land plant evolution. To explore Phe41 function in histone deposition, we generated *Arabidopsis* transgenic plants expressing a series of single amino acid substitutions between H3.1 and H3.3. Our fluorescence microscopy and genomic analyses revealed that while Tyr41 is not important for the H3.3 deposition pattern, Phe41 is critical for H3.1 genomic distribution. Unlike the specific enrichment of H3.1 with silent regions, H3.1F41Y lost this preference and gained ectopic accumulation at actively transcribed regions marked with active histone modifications (H3K36me2 and H3K9ac). Consistently, our reciprocal tail and core domain swap between H3.1 and H3.3 experiments showed that the H3.1 core, while necessary, is insufficient to restrict H3.1 in the silent regions. Collectively, our data show that Phe41 is important for H3.1 global distribution and may act collaboratively with the H3.1 core to regulate its deposition pattern.

## Results

### H3.1Phe41 evolved independently during plant evolution

**Fig. 1:**
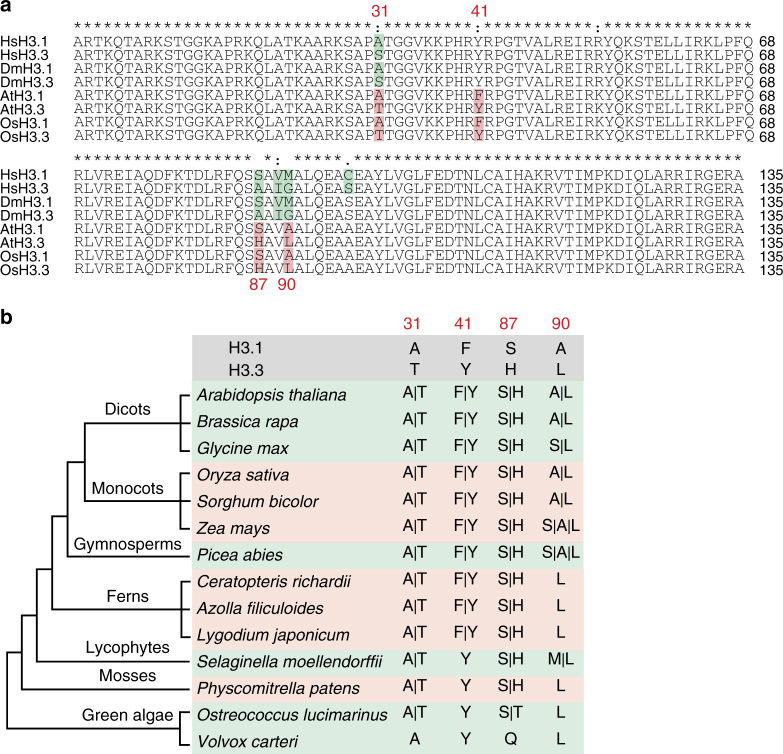
Histone H3 phenylalanine 41 evolved in vascular plants. **a** Alignment of H3.1 and H3.3 amino acid sequences in *Homo sapiens* (Hs), *Drosophila melanogaster* (Dm), *Arabidopsis thaliana* (At), and *Oryza sativa* (Os). The salmon pink color highlights the amino acid differences in plants and the light green color highlights the amino acid differences in animals. **b** Cladogram analysis of H3.1 and H3.3 in different plant species. Amino acid residues at positions 31, 41, 87, and 90 are shown for each species

Histone H3 is known to be highly conserved in plants and metazoans^29,31^. In animals, histone variants H3.1 and H3.3 are typically distinguished at residues 31, 87, 89, and 90. While the difference between H3.1 and H3.3 at positions 31, 87, and 90 is conserved between plants and animals, H3.1 differs from H3.3 by an additional residue at position 41 in the flowering plants *Arabidopsis thaliana* and *Oryza sativa* (Fig. 1a). Unlike Tyr41 that is present in both animal H3 variants, plant H3.1 and H3.3 contain Phe41 and Tyr41, respectively, implying that Phe41 may have evolved independently in plants. To test this idea, we performed BLASTP and TBLASTN searches against the available genomes of dicots and monocots, as well as ancient species such as gymnosperms, ferns, lycophytes, mosses, and green algae using the *Arabidopsis* histone sequence as a reference (Supplementary Data 1). We noted that two H3 variants were generally found in all land plants, including angiosperms, gymnosperms, ferns, lycophytes, and mosses (Fig. 1b), which is consistent with previous findings^30,31^. Interestingly, while the green algae *Ostreococcus* has two forms of H3, we found only a single form of H3 in *Volvox* (another green algae) (Fig. 1b). These data suggest that H3 variants likely evolved from the ancestor of green algae. We also found that, although substitutions at positions 31 and 87 were present since the divergence of H3 variants, Phe41 first appeared in H3.1 in ferns (about 300 million years ago) and became stable during land plant evolution (Fig. 1b and Supplementary Data 1). This vascular-plant-specific Phe41 residue localizes at the amino terminus of the first helix of H3 (the aN1-helix) where DNA enters the nucleosome (Supplementary Fig. 1)^2,32^. This specific feature, together with its high conservation among seed plants, indicates that Phe41 may have evolved to play a role in vascular-plant-specific chromatin regulation.

### Tyr41 is dispensable for H3.3 genomic distribution pattern

**Fig. 2:**
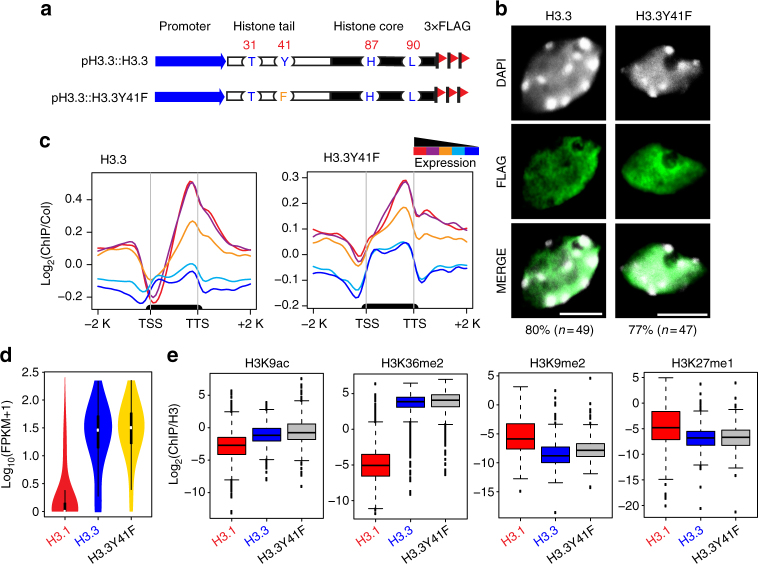
Tyr41 is dispensable for H3.3 genomic distribution. **a** Schematic diagram of wild-type H3.3 and H3.3Y41F FLAG-tagged constructs. **b** Localization of wild-type H3.3 and H3.3Y41F protein in nuclei (bar = 5 μm). The *n* represents the total number of examined nuclei. The percentage describes the ratio of the nuclei showing the H3 distribution pattern out of total examined nuclei. DAPI indicates the DAPI staining of the nucleus. **c** Metagene plots of wild-type H3.3 and H3.3Y41F ChIP-seq reads over five groups of genes divided based on their expression levels. The black bar in the *x*-axis represents genes. TSS, transcription start sites; TTS, transcription terminal sites; −2 K and +2 K represent 2 kb upstream of TSS and 2 kb downstream of TTS, respectively. The *y*-axis represents the log_2_ value of H3.3 and H3.3Y41F ChIP-seq reads normalized to those of Col-0. **d** Violin plot showing the expression levels of genes associated with H3.1, H3.3, and H3.3Y41F ChIP-seq peaks. The *y*-axis represents the log_10_ value of FPKM +1. FPKM, fragments per kilobase of transcript per million mapped reads. RNA-seq data were from 3-week-old seedlings. **e** Boxplots of histone modification levels over ChIP-seq peaks of H3.1, H3.3, and H3.3Y41F. The *y*-axis represents the ChIP-seq read density normalized by H3. Histone modification ChIP-seq data were from 2-week-old aerial tissues

The unique difference of residue 41 between plant H3.1 and H3.3 led us to examine its potential function. We first investigated whether Tyr41 is important for H3.3 localization. To this end, we generated a transgene construct containing a single mutation of H3.3Y41 to phenylalanine (H3.3Y41F) fused with a C-terminal 3×FLAG epitope tag driven by its native promoter (Fig. 2a). As a control, we used 3×FLAG-tagged wild-type H3.3 transgenic plant (named H3.3) that have no phenotypic differences from the non-transgenic wild-type Columbia-0 (Col-0) as described previously^33^. The H3.3Y41F showed similar expression levels to that of H3.3 (Supplementary Fig. 2a). We next determined the subcellular localization of H3.3Y41F by immunofluorescence microscopy. As expected and consistent with previous reports^28,34,35^, 80% of wild-type H3.3 (39 out of 49 nuclei) localized in the nucleoplasm with little or no overlap of 4′,6-diamidino-2-phenylindole (DAPI)-stained heterochromatic chromocenters (Fig. 2b and Supplementary Data 2). Similarly, H3.3Y41F protein localized in the nucleoplasm as a diffuse pattern with no noticeable signals in chromocenters in approximately 77% (36 out of 47) of examined nuclei (Fig. 2b and Supplementary Data 2).

To assess the genome-wide localization of H3.3Y41F at high resolution, we performed chromatin immunoprecipitation followed by massively parallel sequencing (ChIP-seq). We first examined the relationship between H3.3Y41F distribution and gene expression activity by classifying a total of 28,000 genes of the *Arabidopsis* genome into five groups based on their expression levels (Supplementary Fig. 2b). Plotting the ChIP-seq reads over these gene groups revealed a positive correlation between H3.3Y41F and gene expression levels, similar to the wild-type H3.3 pattern (Fig. 2c). Additionally, the majority of H3.3Y41F-enriched genes were actively expressed (Fig. 2d), demonstrating that H3.3Y41F is associated with active transcription. We next tested whether H3.3Y41F was associated with specific histone modifications. First, we examined the overall histone modification levels in wild-type and H3 mutant transgenic plants and found no noticeable differences of H3K9ac, H3K36me3, H3K9me2, and H3K4me3 levels in H3.3 and H3.3Y41F transgenic plants (Supplementary Fig. 2c). We then plotted various histone modifications on H3.3Y41F peaks and found that H3.3Y41F peaks were enriched over active marks (H3K9ac and H3K36me2) and depleted in two repressive marks H3K9me2 and H3K27me1 (Fig. 2e), consistent with their association with actively transcribed regions. Together, the similar subcellular localization and genomic distribution patterns of H3.3Y41F and wild-type H3.3 suggest that Y41 is dispensable for H3.3 distribution.

### Phe41 is important for global H3.1 distribution

**Fig. 3:**
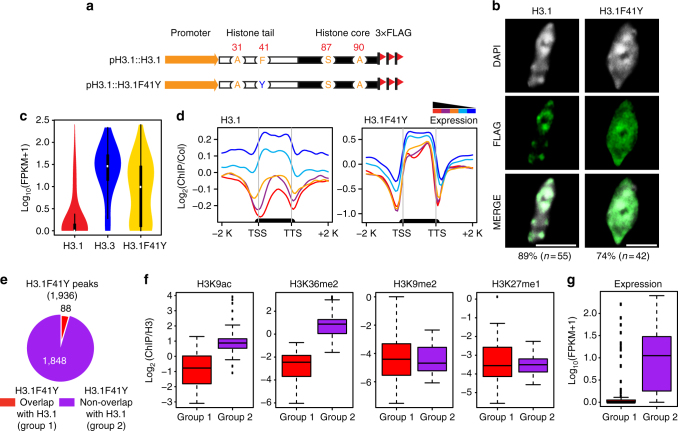
Phe41 plays an important role in H3.1 distribution. **a** Schematic diagram of wild-type H3.1 and H3.1F41Y FLAG-tagged constructs. **b** Localization of wild-type H3.1 and H3.1F41Y protein in nuclei (bar = 5 μm). The *n* represents the total number of examined nuclei. The percentage describes the ratio of the nuclei showing the H3 distribution pattern out of total examined nuclei. DAPI indicates the DAPI staining of the nucleus. **c** Violin plot of the expression levels of genes associated with H3.1, H3.3, and H3.1F41Y ChIP-seq peaks. The *y*-axis represents the log_10_ value of FPKM +1. RNA-seq data were from 3-week-old seedlings. **d** Metagene plots of wild-type H3.1 and H3.1F41Y ChIP-seq reads over five groups of genes divided based on their expression levels. The black bar in the *x*-axis represents genes. TSS transcription start sites; TTS transcription terminal sites; −2 K and +2 K represent 2 kb upstream of TSS and 2 kb downstream of TTS, respectively. The *y*-axis represents the log_2_ value of H3.1 and H3.1F41Y ChIP-seq reads normalized to those of Col-0. **e** Pie chart showing the H3.1F41Y ChIP-seq peaks that overlapped with H3.1 peaks (defined as Group 1) or not overlapped with H3.1 peaks (H3.1F41Y unique peaks, defined as Group 2). **f** Boxplots of histone modification levels over peak-associated genes from the two groups defined in **e**. The *y*-axis represents the ChIP-seq reads normalized to those of H3. Histone modification ChIP-seq data were from 2-week-old aerial tissues. **g** Boxplot of expression levels of genes associated with the peaks from the two groups defined in **e**. FPKM, fragments per kilobase of transcript per million mapped reads

To examine the importance of the vascular-plant-specific H3.1F41, we similarly generated C-terminal 3×FLAG-tagged H3.1 containing a single mutation of H3.1F41 to tyrosine (H3.1F41Y) driven by its native promoter (Fig. 3a), which had equivalent protein expression levels as those of epitope-tagged wild-type H3.1 (Supplementary Fig. 2a). Distinct from the preferential localization of H3.1 in chromocenters, we found that H3.1F41Y protein signals were present not only in chromocenters but also in the nucleoplasm in 74% of the examined nuclei (Fig. 3b and Supplementary Data 2). This result suggested that residue Phe41 might play a role in H3.1 distribution.

To directly and precisely determine its genome-wide distribution pattern, we performed ChIP-seq analysis in H3.1F41Y. Consistent with our cytological data (Fig. 3b), we noted that while H3.1 is mostly associated with silent genes, H3.1F41Y showed a comparable enrichment at both active and silent genes (Fig. 3c). This trend became much clearer when we examined the H3.1F41Y distribution pattern on five gene groups classified by their expression levels (Fig. 3d). The same distribution pattern was also observed in our H3.1F41Y ChIP-seq replicates (Supplementary Fig. 3a). Consistently, quantitative PCR analysis from two independent ChIP experiments of H3.1, H3.3, H3.1F41Y, and H3.3Y41F showed a specific enrichment of H3.3Y41F at active genes and an enrichment of H3.1F41Y at both active genes and silent genes (Supplementary Fig. 3b, c). To further investigate specific features of these ectopic enrichment regions of H3.1F41Y, we identified a total of 1,936 regions significantly enriched for H3.1F41Y (*p* < 1e−3). Compared to H3.1, 1,848 peaks (95%) were uniquely present in H3.1F41Y and did not overlap with H3.1-enriched peaks (Fig. 3e). We next examined the histone modifications over the genes associated with H3.1F41Y unique peaks. We found that these genes showed high levels of active marks (H3K9ac and H3K36me2) and low enrichment in H3K9me2 and H3K27me1 silent marks (Fig. 3f). In addition, the new H3.1F41Y enrichment peaks tended to associate with highly expressed genes (Fig. 3g).

Taken together, our cytological and genomic data revealed that, unlike the specific association of H3.1 with silent regions, H3.1F41Y lost this preference and was enriched at both active and silent regions. This unexpected distribution pattern indicates that amino acid Phe41 of H3.1 plays an important role in its genomic localization.

### Ala/Thr31 is not decisive for H3 distribution patterns

**Fig. 4:**
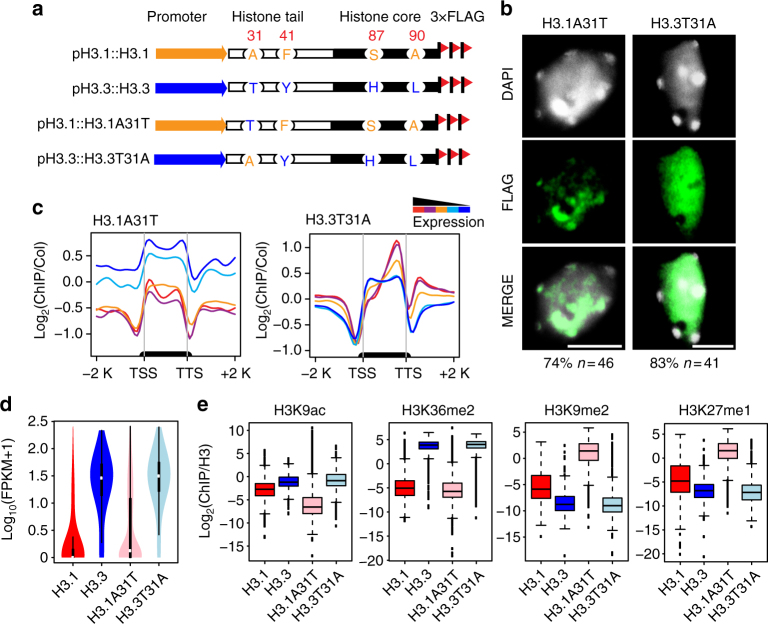
Ala/Thr31 is not decisive for genome-wide H3 distribution pattern. **a** Schematic diagram of H3.1, H3.3, H3.1A41T, and H3.3T31A FLAG-tagged constructs. **b** Localization of H3.1A31T and H3.3T31A protein in nuclei (bar = 5 μm). The *n* represents the total number of examined nuclei. The percentage describes the ratio of the nuclei showing the H3 distribution pattern out of total examined nuclei. DAPI indicates the DAPI staining of the nucleus. **c** Metagene plots of H3.1A31T and H3.3T31A ChIP-seq reads over five groups of genes divided based on their expression levels. The black bar in the *x*-axis represents genes. TSS transcription start sites, TTS transcription terminal sites; −2 K and +2 K represent 2 kb upstream of TSS and 2 kb downstream of TTS, respectively. The *y*-axis represents the log_2_ value of H3.1A31T and H3.3T31A ChIP-seq reads normalized to those of Col-0. **d** Violin plots of the expression levels of genes associated with H3.1, H3.3, H3.1A31T, and H3.3T31A ChIP-seq peaks. The *y*-axis represents the log_10_ value of FPKM +1. FPKM fragments per kilobase of transcript per million mapped reads. RNA-seq data were from 3-week-old seedlings. **e** Boxplots of histone modification levels in ChIP-seq peaks of H3.1, H3.3, H3.1A31T, and H3.3T31A. The *y*-axis represents the ChIP-seq reads normalized with those of H3. Histone modification ChIP-seq data were from 2-week-old aerial tissues

Besides position 41, H3.1 and H3.3 also differ at residue 31 in the N-terminal tail. To examine whether the difference in position 31 plays a role in histone distribution, we generated single amino acid substitutions H3.1A31T and H3.3T31A fused with a 3×FLAG tag driven by their respective promoters (Fig. 4a). Immunofluorescence microscopy showed that when alanine at position 31 of H3.1 is mutated to threonine, H3.1A31T localization remains largely the same as the wild-type H3.1 (Fig. 4b and Supplementary Data 2). Similarly, T31A substitution of H3.3 did not affect its subcellular localization compared to the wild-type H3.3 (Fig. 4b and Supplementary Data 2). Plotting the ChIP reads over different gene expression groups showed that H3.1A31T was negatively correlated with gene activity and H3.3T31A was enriched at active genes (Fig. 4c, d). In addition, H3.1A31T-enriched and H3.3T31A-enriched regions showed similar correlations with histone modification marks as wild-type H3.1 and H3.3 (Fig. 4e), suggesting that residue 31 is not decisive for H3 deposition.

### Both Phe41 and histone core determine H3.1 distribution

**Fig. 5:**
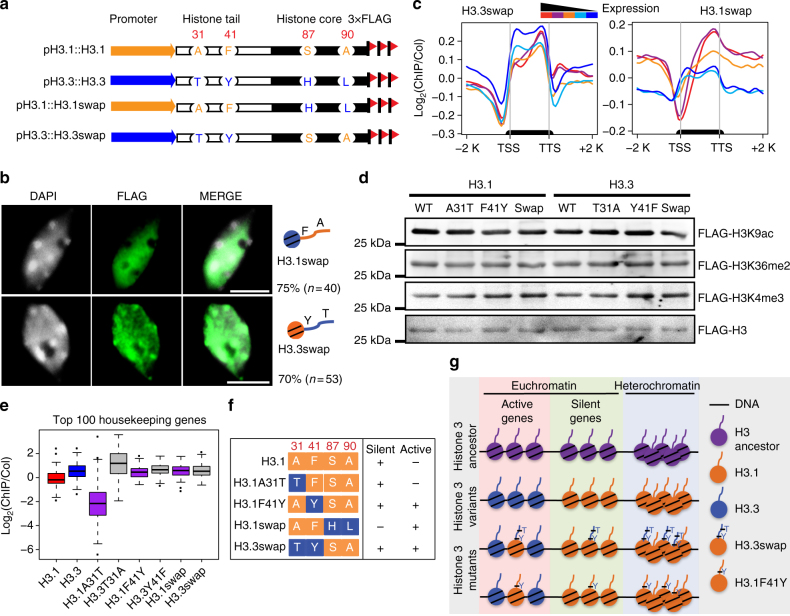
Phe41 coordinates with the histone core region to determine H3.1 distribution pattern. **a** Schematic diagram of H3.1, H3.3, H3.1swap, and H3.3swap FLAG-tagged constructs. **b** Localization of H3.1swap and H3.3swap protein in nuclei (bar = 5 μm). The *n* represents the total number of examined nuclei. The percentage describes the ratio of the nuclei showing the H3 distribution pattern out of total examined nuclei. DAPI indicates the DAPI staining of the nucleus. **c** Metagene plots of H3.1swap and H3.3swap ChIP-seq reads over five groups of genes divided based on their expression levels. The black bar in the *x*-axis represents genes. TSS transcription start sites, TTS transcription terminal sites; −2 K and +2 K represent 2 kb upstream of TSS and 2 kb downstream of TTS, respectively. The *y*-axis represents the log_2_ value of H3.1swap and H3.3swap ChIP-seq reads normalized to those of Col-0. **d** FLAG-tagged wild-type and mutant H3 proteins have similar post-translational modifications. Immunoblotting showing similar H3K9ac, H3K36me2, and H3K4me3 levels from transgenic plants expressing FLAG-tagged H3 variants. FLAG-tagged H3 proteins were first purified with anti-FLAG beads, and then immunoblotted with respective antibodies to determine the histone modification levels. **e** Boxplot shows the log_2_ value of ChIP-seq reads of wild-type and mutant H3 normalized to those of Col-0 over the top 100 highly expressed housekeeping genes. **f** Summary of the genomic distribution patterns of H3.1, H3.3, and their respective mutants. **g** A working model for Phe41 function in H3.1 distribution. The yeast and green algae contain one single H3 (likely the H3 ancestor) that serves as a substrate for both replication-dependent and replication-independent deposition pathways for nucleosome assembly. In vascular plants, F41 of H3.1 may play an important role in histone deposition and/or histone replacement by restricting H3.1 in the silent and heterochromatic regions. H3.3swap and H3.1F41Y mutation may prevent this restriction resulting in the accumulation of H3.1 in both active and silent genes

Previous studies have revealed critical roles of amino acids within the histone core region in determining histone deposition in both plants and animals^27,28,36^. Our unexpected findings that the genome-wide pattern of H3.1 enrichment is also dependent on residue Phe41 of the histone tail directed our investigation on the potential crosstalk between the “histone tail” and “histone core” in H3 deposition. To this end, we performed a reciprocal tail and core domain swap between H3.1 and H3.3 to create H3.1swap (H3.1 tail with H3.3 core) and H3.3swap (H3.3 tail with H3.1 core) (Fig. 5a). These swapped genes were fused to a C-terminal 3×FLAG epitope tag and were driven by their respective endogenous promoters. In line with previous studies that core residues of H3.3 are important for its subcellular localization^28^, H3.1swap protein showed a similar distribution pattern to that of wild-type H3.3. Furthermore, H3.1swap protein showed a diffused distribution pattern in the nucleoplasm and did not expand within the densely DAPI-stained chromocenters in approximately 75% of the tested nuclei (Fig. 5b and Supplementary Data 2). Those results suggest that the H3.3 core was sufficient to guide the histone incorporation into active genomic regions. Interestingly, the H3.3swap was present in the nucleoplasm as well as in the chromocenters, unlike wild-type H3.1 (Fig. 5b and Supplementary Data 2). Consistently, H3.1swap was found to be enriched at the body of active genes with a strong bias toward the 3′ end (Fig. 5c). Three biologically independent ChIP-seq experiments of H3.3swap showed similar distribution patterns (Fig. 5c and Supplementary Fig. 4a). We further confirmed the pattern by performing quantitative PCR analysis with two independent ChIP products of H3.1swap and H3.3swap and found that H3.1swap was enriched at active genes while H3.3swap was enriched at both active genes and silent genes (Supplementary Fig. 4b, c). These data suggest that the H3.1 core is required but insufficient to restrict H3.1 in the silent regions. The ectopic distribution pattern caused by F41Y mutation led us to examine whether the modifications on the tails of FLAG-tagged H3 mutants were affected. To this end, we first purified the FLAG-tagged wild-type and mutant H3 with an anti-FLAG antibody and then examined the co-enrichment of histone marks by immunoblotting. We did not observe a notable difference of H3K9ac, H3K36me2, and H3K4me3 levels between FLAG-tagged wild-type and mutant H3 (Fig. 5d), suggesting that the overall modifications of FLAG-tagged H3 were not affected by the F41Y mutation.

To further confirm the distribution patterns of H3 mutants, we plotted the ChIP-seq reads of different mutants over active or silent chromatin states defined previously^37^. We found that H3.1F41Y and H3.3swap were enriched in both the active and the silent chromatin states (Supplementary Fig. 4d). Consistently, both H3.1F41Y and H3.3swap were enriched in the top 100 highly expressed *Arabidopsis* housekeeping genes^38^ (Fig. 5e and Supplementary Fig. 4e). The similar distribution patterns of H3.1F41Y and H3.3swap (Fig. 5f) reinforced our view that Phe41 of the H3.1 tail is important and may act collaboratively with H3.1 core to regulate histone distribution patterns.

## Discussion

Histone variants H3.1 and H3.3 differ only in four or five amino acids, yet they have distinct deposition patterns and chromatin function^8–10^. The canonical variant H3.1 is predominately expressed in the S phase and incorporated into chromatin in a DNA replication-coupled pathway (RC), while H3.3 is expressed and deposited via a DNA replication-independent (RI) manner throughout the cell cycle^27^. Studies from both plants and animals have established that the key amino acid residues within the histone core region are critical for histone deposition and the residues that lie in the N-terminal tail are subject to versatile PTMs for function^9,27,28^. In this study, we demonstrate a previously unexplored role of the histone tail in the genome-wide histone deposition. The variation of position 41 between H3.1 and H3.3 is unique and conserved in vascular plants^30,31^. Our results showed that, unlike H3.1 that is preferentially enriched at silent regions, H3.1F41Y was found at both silent and actively transcribed regions. These findings support a model wherein Phe41 of H3.1 may play an important role in histone deposition and/or histone replacement of H3.1 by H3.3 at the active genes (Fig. 5g).

The precise mechanism of how H3.1F41Y was enriched at active genomic regions is not known. Several possibilities could account for this distribution pattern. First, Phe41 of H3.1 may play an important role in mediating histone deposition. The yeast and green algae contain one single H3 (likely the H3 ancestor) that serves as a substrate for both RC and RI deposition pathways. Somehow this ability to serve as a substrate for RI is lost during plant H3.1 evolution. It is possible that F41 of H3.1 prevents the incorporation of H3 into the RI pathway and that the H3.1F41Y mutation may be able to be deposited by both RC and RI pathways resulting in the accumulation of H3.1 in both active and silent regions. Second, H3.3 (also known as “replacement histone variant”) replaces canonical H3.1 at actively transcribed regions during transcription^8,39,40^. It is possible that the continuous exchange of H3.1 by H3.3 requires Phe41 and that the F41Y mutation prevents this replacement resulting in the accumulation of H3.1F41Y in active genomic regions. Third, Phe41 may serve as an important recognition site for certain heterochromatin factors and F41Y mutation may block this binding, thus preventing it from being held in heterochromatic regions. It is well established that histone tails are subject to various PTMs^15,25,41–43^. A fourth possibility is that Phe41 of H3.1 and Tyr41 of H3.3 may undergo distinct PTMs that provide docking stations for specific histone chaperones or chromatin remodeling factors for histone deposition. Based on our initial investigation of the three histone marks (H3K9ac, H3K36me2, and H3K4me3), we did not observe any significant differences in their overall levels between FLAG-tagged wild-type and mutant H3 immuno-purified by an anti-FLAG antibody (Fig. 5d). We also noted similar chromatin incorporation patterns between wild-type and mutant H3 proteins (Supplementary Fig. 5). Although Tyr is biochemically similar to Phe, a distinct feature of Tyr is the presence of a hydroxyl group that is bonded to its benzene ring, which allows it to be potentially phosphorylated^32,44,45^. Consistent with this idea, Tyr41 of H3 is phosphorylated (H3Y41ph) by the activation of Janus kinase (JAK) in human cells and H3Y41ph is specifically associated with promoters and active regions by preventing heterochromatin protein 1α from binding to H3^32,46^. Hence, we hypothesize that *Arabidopsis* H3.1F41Y may similarly enable the phosphorylation, prevent some heterochromatin proteins binding, and thus redirecting H3.1 to the active regions. The fact that no JAK homolog has been found in *Arabidopsis* and other plants^47^ raises the interesting possibility that Phe41 may have evolved to distinguish H3.1 from H3.3 when Tyr41 in H3.3 was unable to be phosphorylated in plants. In the future, it will be interesting to determine whether H3.3Tyr41 is phosphorylated and whether there is a yet-to-be identified protein kinase responsible for Tyr41 phosphorylation in plants.

The crucial roles of histone chaperones in histone variant deposition have been extensively investigated^9,10,13,48,49^. Anti-silencing factor-1 (ASF1) and chromatin assembly factor (CAF-1) were found to be present in human canonical H3.1 pre-deposition complexes and histone regulator A (HIRA) in H3.3 pre-deposition complexes^39,48^. Ubinuclein-1 of HIRA complex binds Gly90 and confers the H3.3-specific-binding^50^. The death domain-associated protein (DAXX) was also identified as an H3.3-specific chaperone by recognizing Gly90^51,52^. Interestingly, while ASF1, CAF-1, and HIRA orthologs have been found in *Arabidopsis*, the corresponding orthologs of human DAXX are missing in plants^53^. In contrast to the vertebrate cells where ASF1 and CAF-1 depletion results in cell death^54,55^, the *Arabidopsis* loss-of-function mutants of CAF-1 and ASF1 are viable with minor growth retardation and developmental defects^56–59^. Similarly, while mammalian HIRA is required for fertilization and early development^60,61^, *Arabidopsis* HIRA mutants do not perturb sexual reproduction and embryogenesis^34,62^. These distinct behaviors indicate that histone chaperones might function differently in plants and animals. The unexpected enrichment of H3.1F41Y at active regions prompts us to speculate that a vascular-plant-specific chaperone co-evolved with Phe41 to facilitate its deposition. We attempted to identify the histone chaperones associated with H3.1F41 and H3.1F41Y by immunoprecipitation and mass spectrometry. However, despite extensive efforts, we have been unable to isolate a sufficient amount of peptides corresponding to any known H3.1 chaperones. This was largely due to the fact that only approximately 1% of total histones were from epitope-tagged transgenic H3 proteins and 99% were from endogenous H3 as observed in our previous study^33^. Similarly, a previous study using GFP-tagged H3.1 and H3.3 in *Drosophila* also showed that each of the fusion proteins constituted <0.5% of the total H3 in cells^36^.

Our cladogram analysis revealed that Phe41 first appeared in H3.1 in ferns and became stable during seed plant evolution. This vascular-plant-specific residue Phe41 is important for H3.1 genome-wide distribution (Fig. 5f). The implication for these observations is twofold. First, given that metazoan H3.1 and H3.3 have the same Tyr at the position 41, Phe41 may have evolved to accommodate certain plant-specific needs during development. Ancestor plant species, such as green algae and mosses, only contain Tyr41 in all H3 variants, and the Tyr and Phe difference in the two histone variants was first found in ferns (Fig. 1b). Compared to mosses, gymnosperms and flowering plants are more complex in tissues and cell types along with seed producing. Second, as an immobile organism, plants need to develop an effective strategy to adjust their transcription and chromatin landscape promptly to adapt and survive in adverse conditions. The development of Phe41 in H3.1 might be a more specialized means of accomplishing these processes by adding another layer of distinction to the H3.3 variant during plant evolution. Although the functional importance of Phe41 in H3.1 genome-wide distribution has been established in this study, its precise deposition mechanism remains an important challenge for future work.

In summary, we have characterized the function of vascular-plant-specific Phe41 and how it may act collaboratively with the key residues in the H3.1 core region to ultimately determine its distribution pattern. Our results provide an important insight into the evolutionary significance of H3.1Phe41 and suggest that H3.1Phe41 may have evolved to provide another layer of histone deposition regulation in plants.

## Methods

### Plant materials and constructs

*Arabidopsis thaliana* ecotype Col-0 was used and grown in soil at 21 °C under constant light. The genomic DNA of H3.1 (AT5G10390) and H3.3 (AT4G40040) with their 1 kb promoters was amplified and cloned into pENTR/D-TOPO (Thermo Fisher Scientific). Single amino acid substitution was generated by site-directed mutagenesis using the respective pENTR/D plasmid as a backbone (see primers in Supplementary Data 3). For H3.1swap and H3.3swap constructs, histone tail and core DNA fragments were amplified separately, and the histone tail fragments were combined with swapped histone core fragments by overlapping PCR. The resulting pENTR/D plasmid constructs were recombined into pEarleyGate302 binary vectors^63^ to create 3xFLAG-tagged fusion proteins. Upon confirmation with Sanger sequencing, the constructs were transformed into Col-0 by Agrobacterium-mediated transformation^64^.

### Immunofluorescence

An immunofluorescence experiment was performed as previously described^65^. Briefly, isolated nuclei were diluted in sorting buffer (100 mM Tris-HCl, pH 7.5, 50 mM KCl, 2 mM MgCl_2_, 0.05% Tween-20, 5% sucrose) and then spotted on a poly-lysine-coated slide. The slides were air dried and fixed with 4% formaldehyde in potassium phosphate-buffered saline (KPBS) (128 mM NaCl, 2 mM KCl, 8 mM Na_2_HPO_4_, 2 mM KH_2_PO_4_, pH 7.2) containing 1% Triton X-100 for 20 min. After washing with KPBS containing 1% Triton for three times, slides were blocked with blocking solution (1% bovine serum albumin in KPBS with 1% Triton) and incubated at 37 °C for 30 min. The blocking solution was washed off with KPBS with 1% Triton and the slides were incubated with an anti-FLAG antibody (Sigma, F3165) with 1:200 dilutions at 4 °C overnight. After washing with KPBS, slides were blocked and incubated with a fluorescent-labeled secondary antibody with 1:200 dilution (LI-COR, 926-49010) at 37 °C for 2 h. After washing with KPBS two times, nuclei were stained with 5 µg/mL DAPI and covered with a coverslip followed by microscope (NIKON ECLiPSE Ti-E) detection.

### ChIP followed with qPCR and sequencing

ChIP was performed as previously described^66^. Briefly, crosslinked nuclei were isolated from 2 g of 3-week-old seedlings with 25 mL nuclear isolation buffer (10 mM HEPES, pH 8, 1 M sucrose, 5 mM KCl, 5 mM EDTA, 0.6% Triton X-100, 0.4 mM PMSF, 1 µg/µL pepstain, and protease inhibitor cocktail tablet; Roche Applied Science) and washed with washing buffer (0.25 M sucrose, 10 mM Tris-HCl, pH 8, 10 mM MgCl_2_, 1% Triton X-100, 1 mM EDTA, 5 mM β-mercaptoethanol, 0.4 mM PMSF, and protease inhibitor cocktail tablet). Chromatin was sheared by sonication and incubated with anti-FLAG M2 magnetic beads (Sigma, M8823) overnight at 4 °C. Purified protein–DNA complex was reverse crosslinked and treated with protease and RNase. DNA was purified with phenol–chloroform method and used for qPCR or sequencing. QPCR was performed with SYBR Green Master Mix using CFX96 Real-Time System 690 (Bio-Rad). Primers are listed in Supplementary Data 3. For sequencing, ChIP libraries were constructed using the Ovation Ultralow Library kit (NuGEN, Part No. 0330) following the manufacturer instructions. The libraries were sequenced with single-end 1 × 50 bp by using the HiSeq 2000 Sequencing System (Illumina) at the UW-Madison Biotechnology Center.

### Sequence identification

*Arabidopsis thaliana* protein sequences were used as queries for BLASTP searches against *Brassica rapa*,* Glycine max, Oryza sativa*,* Sorghum bicolor*,* Zea mays*,* Selaginella moellendorffii*,* Physcomitrella patens*, * Ostreococcus lucimarinus, Volvox carteri* genomes (JGI Phytozome v11.0), and *Picea abies* genome (http://congenie.org)^67^. For searches in fern genomes (*Ceratopteris richardii*,* Azolla filiculoides*, and* Lygodium japonicum*), TBLASTN was used in the NCBI TSA database (https://www.ncbi.nlm.nih.gov/genbank/tsa/). Protein details for each species are listed in Supplementary Data 1. Accession numbers of each protein were obtained from the respective databases.

### Genomic data analysis

Sequencing reads were mapped to Arabidopsis TAIR10 genome with Bowtie 2 (v2.1.0)^68^ using default parameters. Reads mapping to identical positions in the genome were collapsed into one read. ChIP-seq experiments of H3.1F41Y and H3.3swap were repeated two and three times, respectively. Each biological replicate was analyzed independently. Given the similar distribution patterns in each replicate (Supplementary Figs. 3a and 4a), the ChIP-seq replicated files were merged into one single file for further analysis. The total reads obtained for each replicate are listed in Supplementary Data 4.

For plots of ChIP enrichment over genes, each gene was divided into 20 intervals (5% each interval) separately for the body of the gene, 2 kb upstream of the transcription start sites, and 2 kb downstream of the transcription terminal sites. Enrichment regions of each ChIP were defined using the SICER package^69^ (window size 200; fragment size 200; gap size 200) with Col-0 ChIP-seq as a control (Supplementary Data 4).

Histone modification datasets were obtained from a published study^70^. A log_2_ value of ChIP-seq reads normalized to those of H3 ChIP-seq was calculated for each peak or gene, and the average number of a peak/gene file was used for boxplot. The chromatin state information, the top 100 housekeeping genes, and gene expression levels (FPKM (fragments per kilobase of exon model per million mapped reads)) were obtained from published datasets^37,38,66^. The original data sources are listed in Supplementary Data 5. All plots were generated by the R program (v3.2.3) (http://www.R-project.org).

### Immunoprecipitation of FLAG-tagged H3 proteins

Two grams of leaves were ground into fine powders with liquid nitrogen, which then homogenized with 10 mL IP buffer (50 mM Tris-HCl, pH 7, 150 mM NaCl, 5 mM MgCl_2_, 5% glycerol, 0.1% NP40, 1 mM DTT, 1 mM PMSF, and protease inhibitor cocktail tablet). After centrifugation at 10,000×*g* for 10 min, the supernatant was incubated with anti-FLAG M2 magnetic beads (Sigma, M8823) for 3 h at 4 °C with rotation. The protein–bead complex was washed with IP buffer for three times before boiling with sodium dodecyl sulfate (SDS) loading buffer at 95 °C for 10 min. The supernatant was used for western blot.

### Chromatin fractionation

One gram of leaves was ground into fine powders with liquid nitrogen. Nuclei were isolated using the same method as ChIP. The nuclei were resuspended with 1 mL of buffer A (3 mM EDTA, 0.2 mM EGTA) and rotated at 4 °C for 30 min. After centrifugation at 6,500×*g* for 5 min, the pellet was resuspended with 300 µL buffer B (50 mM Tris-HCl, pH 8, 150 mM NaCl, 0.05% NP40) and rotated at 4 °C for 30 min. Supernatant and pellet were separated by centrifuging at 6,500×*g* for 5 min. The pellet was sequentially extracted using the same method with 300 µL buffer C (50 mM Tris-HCl, pH 8, 500 mM NaCl, 0.05% NP40) and 300 µL buffer D (50 mM Tris-HCl, pH 8, 1 M NaCl, 0.05% NP40). The pellet after buffer D extraction was then resuspended with 300 μl SDS loading buffer before boiling at 95 °C for 10 min.

### Immunoblotting

Nuclei were isolated using the same method as ChIP. Nuclear protein was extracted by boiling nuclei with SDS loading buffer at 95 °C for 10 min. FLAG-tagged proteins were detected by a horseradish peroxidase conjugated with anti-FLAG antibody (Sigma, A8592). The following histone antibodies were used for western blot: H3K9ac (1:5,000 dilution, Millipore, 07-352), H3K4me3 (1:5,000 dilution, Millipore, 04-745), H3K9me2 (1:1,000 dilution, Abcam, ab1220), H3K36me2 (1:5,000 dilution, Abcam, ab9049), H3K36me3 (1:5,000 dilution, Abcam, ab9050), and H3 (1:7,000 dilution, Abcam, ab1791). Western blots were developed using ECL Plus Western Blotting Detection System (GE Healthcare, RPN2132). Raw images of the blots are included in Supplementary Fig 6.

### Data availability

The ChIP-seq datasets have been deposited into the NCBI Gene Expression Omnibus (GEO) database with an accession number GSE93223. All other data supporting the findings of this study are available within the manuscript and its supplementary files or are available form the corresponding author upon request.

## Electronic supplementary material

Supplementary Information

Description of Additional Supplementary Files

Supplementary Data 1

Supplementary Data 2

Supplementary Data 3

Supplementary Data 4

Supplementary Data 5

## References

[1] Kornberg RD (1974). Chromatin structure: a repeating unit of histones and DNA. Science.

[2] Luger K, Mader AW, Richmond RK, Sargent DF, Richmond TJ (1997). Crystal structure of the nucleosome core particle at 2.8 A resolution. Nature.

[3] Talbert PB (2012). A unified phylogeny-based nomenclature for histone variants. Epigenet. Chromatin.

[4] Kamakaka RT, Biggins S (2005). Histone variants: deviants?. Genes Dev..

[5] Allshire RC, Karpen GH (2008). Epigenetic regulation of centromeric chromatin: old dogs, new tricks?. Nat. Rev. Genet..

[6] Witt O, Albig W, Doenecke D (1996). Testis-specific expression of a novel human H3 histone gene. Exp. Cell. Res..

[7] Hake SB, Allis CD (2006). Histone H3 variants and their potential role in indexing mammalian genomes: the “H3 barcode hypothesis”. Proc. Natl. Acad. Sci. USA.

[8] Szenker E, Ray-Gallet D, Almouzni G (2011). The double face of the histone variant H3.3. Cell. Res..

[9] Jiang D, Berger F (2017). Histone variants in plant transcriptional regulation. Biochim. Biophys. Acta.

[10] Filipescu D, Szenker E, Almouzni G (2013). Developmental roles of histone H3 variants and their chaperones. Trends Genet..

[11] Weber CM, Henikoff S (2014). Histone variants: dynamic punctuation in transcription. Genes Dev..

[12] Maze I, Noh KM, Soshnev AA, Allis CD (2014). Every amino acid matters: essential contributions of histone variants to mammalian development and disease. Nat. Rev. Genet..

[13] Mattiroli F, D’Arcy S, Luger K (2015). The right place at the right time: chaperoning core histone variants. EMBO Rep..

[14] Loyola A, Almouzni G (2007). Marking histone H3 variants: how, when and why?. Trends Biochem. Sci..

[15] Jacob Y (2014). Selective methylation of histone H3 variant H3.1 regulates heterochromatin replication. Science.

[16] Mito Y, Henikoff JG, Henikoff S (2005). Genome-scale profiling of histone H3.3 replacement patterns. Nat. Genet..

[17] Goldberg AD (2010). Distinct factors control histone variant H3.3 localization at specific genomic regions. Cell.

[18] Wirbelauer C, Bell O, Schubeler D (2005). Variant histone H3.3 is deposited at sites of nucleosomal displacement throughout transcribed genes while active histone modifications show a promoter–proximal bias. Genes Dev..

[19] Wong LH (2010). ATRX interacts with H3.3 in maintaining telomere structural integrity in pluripotent embryonic stem cells. Genome Res..

[20] Elsasser SJ, Noh KM, Diaz N, Allis CD, Banaszynski LA (2015). Histone H3.3 is required for endogenous retroviral element silencing in embryonic stem cells. Nature.

[21] Stroud H (2012). Genome-wide analysis of histone H3.1 and H3.3 variants in *Arabidopsis thaliana*. Proc. Natl. Acad. Sci. USA.

[22] Shu H (2014). Arabidopsis replacement histone variant H3.3 occupies promoters of regulated genes. Genome Biol..

[23] Wollmann H (2012). Dynamic deposition of histone variant H3.3 accompanies developmental remodeling of the Arabidopsis transcriptome. PLoS Genet..

[24] Vaquero-Sedas MI, Vega-Palas MA (2013). Differential association of Arabidopsis telomeres and centromeres with histone H3 variants. Sci. Rep..

[25] Johnson L (2004). Mass spectrometry analysis of Arabidopsis histone H3 reveals distinct combinations of post-translational modifications. Nucleic Acids Res..

[26] Zhang K, Sridhar VV, Zhu J, Kapoor A, Zhu JK (2007). Distinctive core histone post-translational modification patterns in Arabidopsis thaliana. PLoS ONE.

[27] Ahmad K, Henikoff S (2002). The histone variant H3.3 marks active chromatin by replication-independent nucleosome assembly. Mol. Cell.

[28] Shi L, Wang J, Hong F, Spector DL, Fang Y (2011). Four amino acids guide the assembly or disassembly of Arabidopsis histone H3.3-containing nucleosomes. Proc. Natl. Acad. Sci. USA.

[29] Baxevanis AD, Landsman D (1996). Histone Sequence Database: a compilation of highly-conserved nucleoprotein sequences. Nucleic Acids Res..

[30] Cui J (2015). Genome-wide identification, evolutionary, and expression analyses of histone H3 variants in plants. Biomed. Res. Int..

[31] Waterborg JH (2012). Evolution of histone H3: emergence of variants and conservation of post-translational modification sites. Biochem. Cell Biol..

[32] Dawson MA (2009). JAK2 phosphorylates histone H3Y41 and excludes HP1alpha from chromatin. Nature.

[33] Sanders D (2017). Histone lysine-to-methionine mutations reduce histone methylation and cause developmental pleiotropy. Plant Physiol..

[34] Nie X, Wang H, Li J, Holec S, Berger F (2014). The HIRA complex that deposits the histone H3.3 is conserved in Arabidopsis and facilitates transcriptional dynamics. Biol. Open.

[35] Derkacheva M (2016). H2A deubiquitinases UBP12/13 are part of the Arabidopsis polycomb group protein system. Nat. Plants.

[36] Schwartz BE, Ahmad K (2005). Transcriptional activation triggers deposition and removal of the histone variant H3.3. Genes Dev..

[37] Sequeira-Mendes J (2014). The functional topography of the arabidopsis genome is organized in a reduced number of linear motifs of chromatin states. Plant Cell.

[38] Czechowski T, Stitt M, Altmann T, Udvardi MK, Scheible WR (2005). Genome-wide identification and testing of superior reference genes for transcript normalization in Arabidopsis. Plant Physiol..

[39] Tagami H, Ray-Gallet D, Almouzni G, Nakatani Y (2004). Histone H3.1 and H3.3 complexes mediate nucleosome assembly pathways dependent or independent of DNA synthesis. Cell.

[40] Nakatani Y, Ray-Gallet D, Quivy JP, Tagami H, Almouzni G (2004). Two distinct nucleosome assembly pathways: dependent or independent of DNA synthesis promoted by histone H3.1 and H3.3 complexes. Cold Spring Harb. Symp. Quant. Biol..

[41] Waterborg JH (1990). Sequence analysis of acetylation and methylation in two histone H3 variants of alfalfa. J. Biol. Chem..

[42] Hake SB (2006). Expression patterns and post-translational modifications associated with mammalian histone H3 variants. J. Biol. Chem..

[43] Loyola A, Bonaldi T, Roche D, Imhof A, Almouzni G (2006). PTMs on H3 variants before chromatin assembly potentiate their final epigenetic state. Mol. Cell.

[44] Hunter T (1998). The Croonian Lecture 1997. The phosphorylation of proteins on tyrosine: its role in cell growth and disease. Philos. Trans. R. Soc. Lond. Ser. B.

[45] Dawson MA (2012). Three distinct patterns of histone H3Y41 phosphorylation mark active genes. Cell Rep..

[46] Rui L (2010). Cooperative epigenetic modulation by cancer amplicon genes. Cancer Cell..

[47] Alvarez-Venegas, R. l., De la Pena, C. & Casas-Mollano, J. A. *Epigenetics in Plants of Agronomic Importance: Fundamentals and Applications: Transcriptional Regulation and Chromatin Remodelling in Plants* (Springer, Cham, 2014).

[48] Filipescu D, Muller S, Almouzni G (2014). Histone H3 variants and their chaperones during development and disease: contributing to epigenetic control. Annu. Rev. Cell Dev. Biol..

[49] Campos EI (2015). Analysis of the histone H3.1 interactome: a suitable chaperone for the right event. Mol. Cell.

[50] Ricketts MD (2015). Ubinuclein-1 confers histone H3.3-specific-binding by the HIRA histone chaperone complex. Nat. Commun..

[51] Drane P, Ouararhni K, Depaux A, Shuaib M, Hamiche A (2010). The death-associated protein DAXX is a novel histone chaperone involved in the replication-independent deposition of H3.3. Genes Dev..

[52] Elsasser SJ (2012). DAXX envelops a histone H3.3-H4 dimer for H3.3-specific recognition. Nature.

[53] Zhu Y, Dong A, Shen WH (2013). Histone variants and chromatin assembly in plant abiotic stress responses. Biochim. Biophys. Acta.

[54] Sanematsu F (2006). Asf1 is required for viability and chromatin assembly during DNA replication in vertebrate cells. J. Biol. Chem..

[55] Hoek M, Stillman B (2003). Chromatin assembly factor 1 is essential and couples chromatin assembly to DNA replication in vivo. Proc. Natl. Acad. Sci. USA.

[56] Zhu Y (2011). Arabidopsis homologues of the histone chaperone ASF1 are crucial for chromatin replication and cell proliferation in plant development. Plant J..

[57] Kaya H (2001). FASCIATA genes for chromatin assembly factor-1 in *Arabidopsis* maintain the cellular organization of apical meristems. Cell.

[58] Mozgova I, Mokros P, Fajkus J (2010). Dysfunction of chromatin assembly factor 1 induces shortening of telomeres and loss of 45S rDNA in Arabidopsis thaliana. Plant Cell.

[59] Exner V, Gruissem W, Hennig L (2008). Control of trichome branching by chromatin assembly factor-1. BMC Plant Biol..

[60] Ray-Gallet D (2002). HIRA is critical for a nucleosome assembly pathway independent of DNA synthesis. Mol. Cell.

[61] Rai TS (2011). Human CABIN1 is a functional member of the human HIRA/UBN1/ASF1a histone H3.3 chaperone complex. Mol. Cell. Biol..

[62] Duc C (2015). The histone chaperone complex HIR maintains nucleosome occupancy and counterbalances impaired histone deposition in CAF-1 complex mutants. Plant J..

[63] Du J (2012). Dual binding of chromomethylase domains to H3K9me2-containing nucleosomes directs DNA methylation in plants. Cell.

[64] Clough SJ, Bent AF (1998). Floral dip: A simplified method for Agrobacterium-mediated transformation of *Arabidopsis thaliana*. Plant J..

[65] Chen Q (2013). Structural basis of a histone H3 lysine 4 demethylase required for stem elongation in rice. PLoS Genet..

[66] Lu L, Chen X, Sanders D, Qian S, Zhong X (2015). High-resolution mapping of H4K16 and H3K23 acetylation reveals conserved and unique distribution patterns in *Arabidopsis* and rice. Epigenetics.

[67] Sundell D (2015). The plant genome integrative explorer Resource: PlantGenIE.org. New. Phytol..

[68] Langmead B, Salzberg SL (2012). Fast gapped-read alignment with Bowtie 2. Nat. Methods.

[69] Zang C (2009). A clustering approach for identification of enriched domains from histone modification ChIP-Seq data. Bioinformatics.

[70] Luo C (2013). Integrative analysis of chromatin states in arabidopsis identified potential regulatory mechanisms for natural antisense transcript production. Plant. J..

