# Supplementary material for: The plant-specific histone residue Phe41 is important for genome-wide H3.1 distribution
Source: Nat Commun. 2018 Feb 12;9:630. doi: 10.1038/s41467-018-02976-9 (PMC5809374; doi:10.1038/s41467-018-02976-9)
Supplement: Supplementary file 1 — Supplementary Information [file 41467_2018_2976_MOESM1_ESM.pdf]

Lu et al.

The plant-specific histone residue Phe41 is important for genome-wide H3.1 distribution

Supplementary Fig 1. Visualization of H3Y41 in the nucleosome.

Supplementary Fig 2. Characterization of FLAG-tagged H3 protein levels and overall histone modifications in transgenic plants.

Supplementary Fig 3. Phe41 plays an important role in H3.1 distribution.

Supplementary Fig 4. Phe41 coordinates with the histone core region to determine the H3.1 distribution pattern.

Supplementary Fig 5. FLAG-tagged wild-type and mutant H3 proteins are similarly incorporated into chromatin as native H3.

Supplementary Fig 6. Raw images of the western blots in this study.

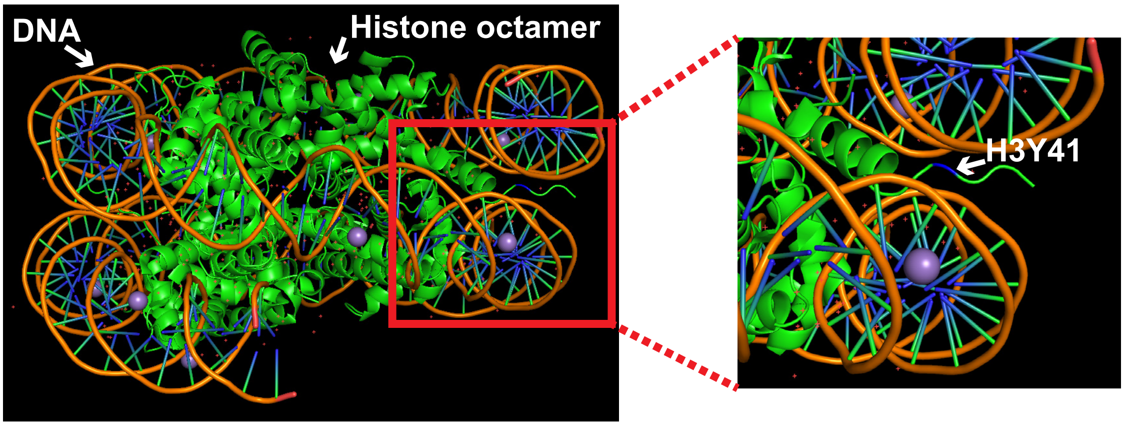

Supplementary Fig 1. Visualization of H3Y41 in the nucleosome.
H3Y41 is highlighted with blue in the right panel. The accession number of the nucleosome structure data is Protein Data Bank (PDB): 2CV5.

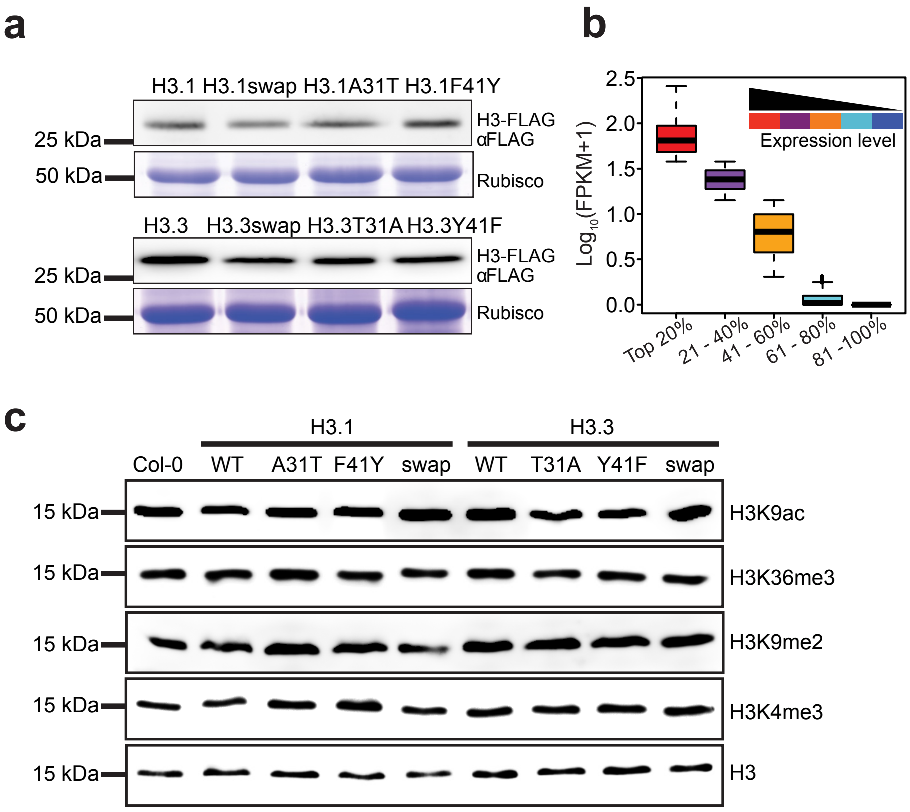

Supplementary Fig 2. Characterization of FLAG-tagged H3 protein levels and overall histone modifications in transgenic plants.
(a) The abundance of FLAG tagged wild-type and mutant H3 proteins in transgenic plants was determined by western blot. Coomassie blue staining of a large subunit of Rubisco was used as a loading control. **(b)** Boxplot of the expression levels of genes divided into five groups based on their expression levels. The top 20% represents the highest expressed gene groups, 81-100% represents the lowest expressed gene groups. The y-axis represents the $\log_{10}$ value of FPKM+1. FPKM, fragments per kilobase of transcript per million mapped reads. **(c)** Western blots of representative histone modification marks in Col-0, wild-type, and mutant H3 transgenic plants.

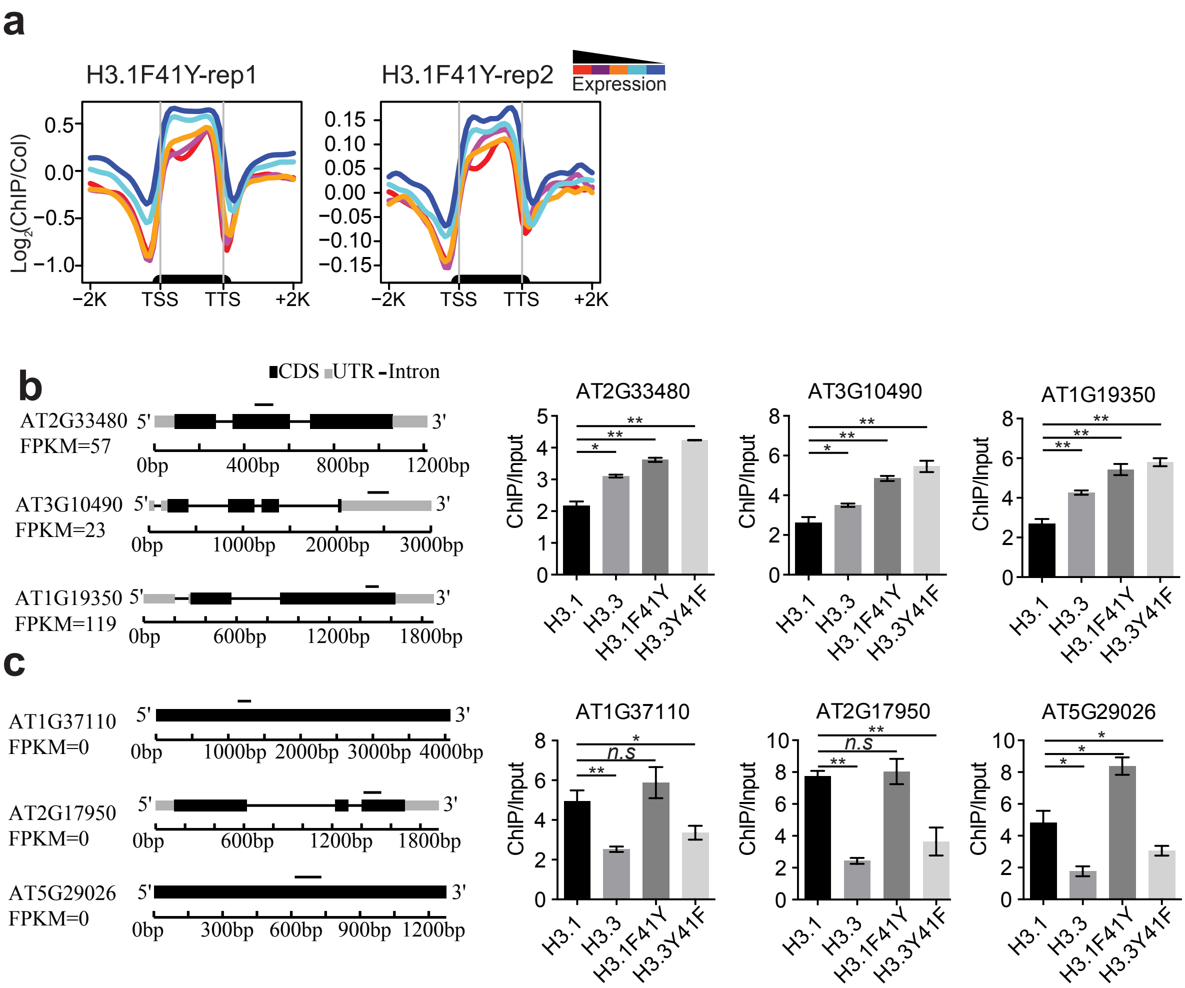

Supplementary Fig 3. Phe41 plays an important role in H3.1 distribution.

(a) Two independent biological replicates of H3.1F41Y ChIP-seq experiments showed similar distribution patterns at both active and silent genes. The y-axis represents the $\log_2$ value of H3.1F41Y ChIP-seq reads normalized to those of Col-0. The black bar in the x-axis represents genes. TSS, transcription start sites; TTS, transcription terminal sites; -2K and +2K represent 2 Kb upstream of TSS and 2 Kb downstream of TTS, respectively. **(b)** ChIP-qPCR showing the enrichment of H3.1F41Y, H3.3Y41F, H3.1, and H3.3 over active genes. **(c)** ChIP-qPCR showing the enrichment of H3.1F41Y, H3.3Y41F, H3.1, and H3.3 over silent genes. Gene structure schematic diagrams are on the left. The black bar indicates the location of the primer. Student's *t*-test, * indicates $p<0.01$; ** indicates $p<0.001$; *n.s* indicates not significant.

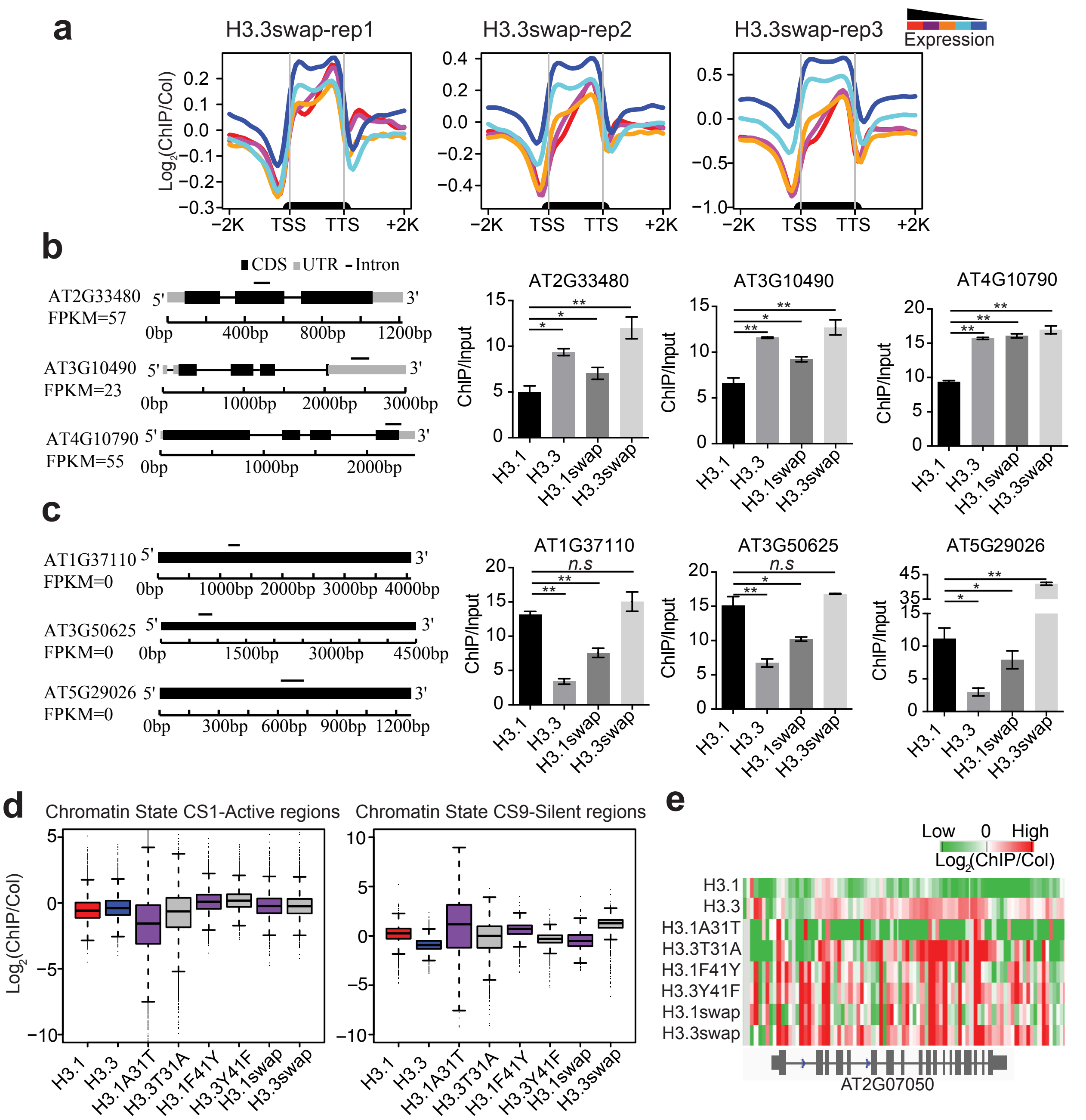

Supplementary Fig 4. Phe41 coordinates with the histone core region to determine the H3.1 distribution pattern.

(a) Three biological replicates of H3.3swap ChIP-seq experiments show similar distribution patterns at both active and silent genes. The y-axis shows normalized H3.3swap ChIP-seq enrichment at five gene groups divided based on their expression levels. The black bar in the x-axis represents genes. TSS, transcription start sites; TTS, transcription terminal sites; -2K and +2K represent 2 Kb upstream of TSS and 2 Kb downstream of TTS, respectively. **(b)** ChIP-qPCR showing the enrichment of H3.1swap, H3.3swap, H3.1, and H3.3 over active genes. **(c)** ChIP-qPCR showing the enrichment of H3.1swap, H3.3swap, H3.1, and H3.3 over silent genes. Gene structure schematic diagrams are on the left. The black bar indicates the location of the primer. Student's *t*-test, * indicates p<0.01; ** indicates p<0.001; *n.s* indicates not significant. **(d)** Boxplots of wild-type and mutant H3 peaks over active (CS1) and silent (CS9) chromatin states. Chromatin states were obtained from published data[37]. **(e)** Representative snapshots of the enrichment of each mutant in the active gene AT2G07050. Color indicates the $\log_2$ value of each ChIP-seq reads normalized to those of Col-0 .

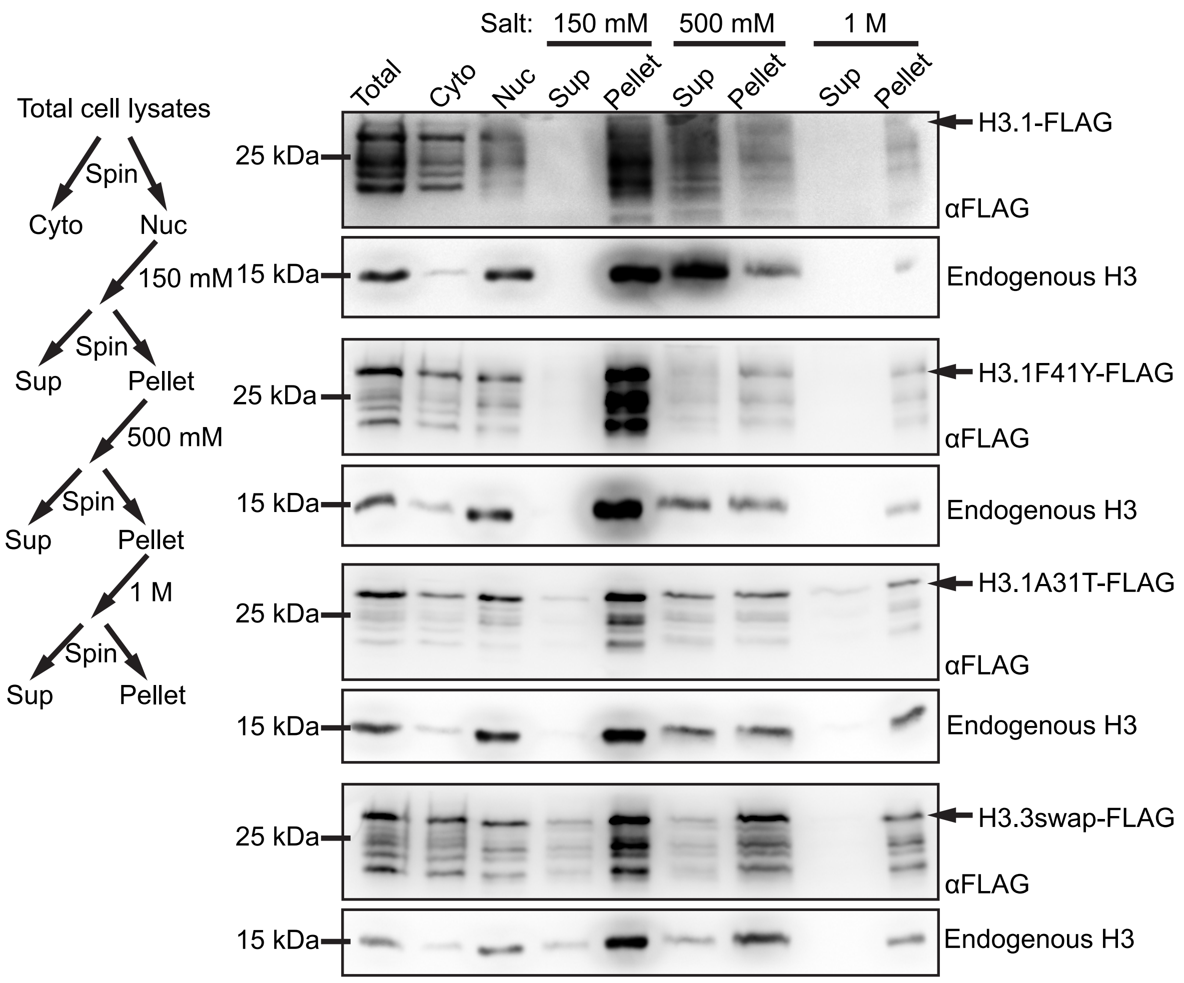

Supplementary Fig 5. FLAG-tagged wild-type and mutant H3 proteins are similarly incorporated into chromatin as native H3.
Nuclei were sequentially extracted with increased salt concentrations (left panel). FLAG-tagged H3 proteins were indicated by arrows. Total, total cell lysates; Cyto, cytoplasm fraction; Nuc, nuclear fraction; Sup, supernatant.

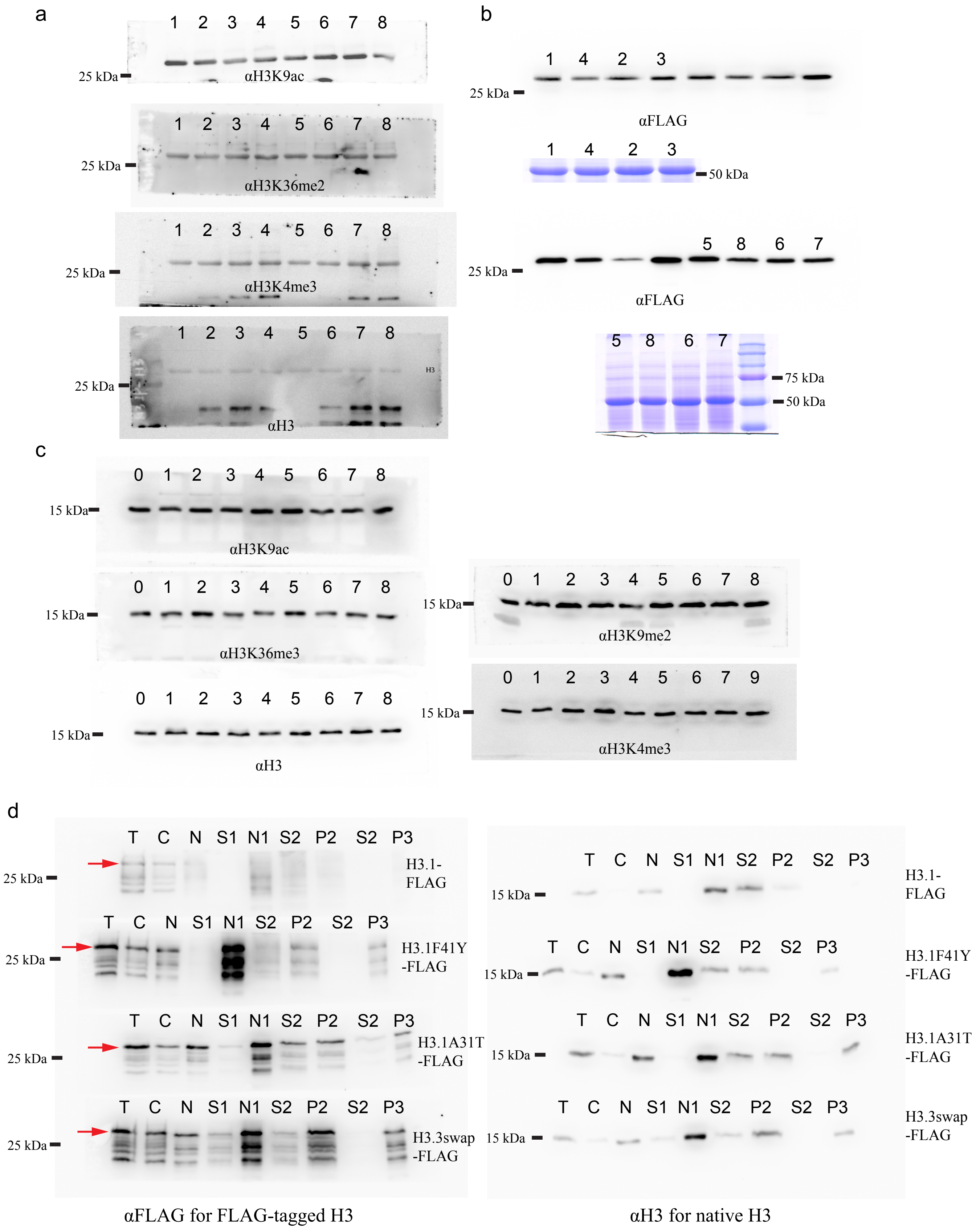

Supplementary Fig 6. Raw images of the western blots in this study.
(a) Raw images of western blots in Fig 5d. 1, H3.1WT; 2, H3.1A31T; 3, H3.1F41Y; 4, H3.1swap; 5, H3.3WT; 6, H3.3T31A; 7, H3.3Y41F; 8, H3.3swap. (b) Raw images of western blots in Supplementary Fig 2a. 1, H3.1WT; 2, H3.1A31T; 3, H3.1F41Y; 4, H3.1swap; 5, H3.3WT; 6, H3.3T31A; 7, H3.3Y41F; 8, H3.3swap. (c) Raw images of western blots in Supplementary Fig 2c. 0, Col-0; 1, H3.1WT; 2, H3.1A31T; 3, H3.1F41Y; 4, H3.1swap; 5, H3.3WT; 6, H3.3T31A; 7, H3.3Y41F; 8, H3.3swap. (d) Raw images of western blots in Supplementary Fig 5. T, total cell lysates; C, cytoplasm fraction; N, nuclear fraction; S1, supernatant of 150 mM NaCl; P1, pellet of 150 mM NaCl; S2, supernatant of 500 mM NaCl; P2, pellet of 500 mM NaCl; S3, supernatant of 1 M NaCl; P3, pellet of 1 M NaCl. Red arrows indicate the FLAG-tagged H3.
