# Supplementary material for: The plant-specific histone residue Phe41 is important for genome-wide H3.1 distribution
Source: Nat Commun. 2018 Feb 12;9:630. doi: 10.1038/s41467-018-02976-9 (PMC5809374; doi:10.1038/s41467-018-02976-9)
Supplement: Supplementary file 2 — Description of Additional Supplementary Files [file 41467_2018_2976_MOESM2_ESM.pdf]

Description of Supplementary Files

File Name: Supplementary Data 1
Description: Amino acid sequences of histone 3 in multiple plant species.

File Name: Supplementary Data 2
Description: Numbers of nuclei examined in immunofluorescence experiments.

File Name: Supplementary Data 3
Description: Primers used in this study.

File Name: Supplementary Data 4
Description: Read and peak numbers for each ChIP-seq experiment.

File Name: Supplementary Data 5
Description: List of original data sources used in this study.
